# Skp2 and Its Emerging Role in the Pathogenesis of Systemic Malignancies Besides Breast Carcinomas

**DOI:** 10.3389/fonc.2012.00134

**Published:** 2012-10-16

**Authors:** Shailendra Kapoor

**Affiliations:** ^1^Mechanicsville, VA, USA

The recent article by Wang et al. (2012a) was highly interesting. Interestingly, recent data suggests that Skp2 expression may play a significant role in the etio-pathogenesis of a number of systemic malignancies besides breast carcinomas.

For instance, the expression of Skp2 in prostate cancers is decreased by androgens and is mediated via pathways that are dependent on p107 as well as pathways that are independent of p107 (Jiang et al., 2012). Similarly, accentuated Skp2 expression in prostate carcinomas may result in the loss of the tumor suppressor gene BRCA2 (Arbini et al., 2011). Thus Skp2 may be a potential therapeutic target for the management of prostate malignancies (Wang et al., 2012b).

Similarly, the prognosis on ovarian cancers is influenced by Skp2. In fact, Skp2 expression by ovarian carcinomas is significantly associated with not only tumor stage but also lymph node metastasis (Lu et al., 2012). Skp2 also is as a receptor for dihydro-testosterone. As a result dihydro-testosterone regulates p27 degradation in ovarian carcinomas (Shi et al., 2011).

Similarly, resistance toward tumor necrosis factor related apoptosis inducing ligand (TRAIL) induced apoptosis in gastrointestinal malignancies especially pancreatic carcinomas is mediated by Skp2 (Schuler et al., 2011). This makes Skp2 a potential onco-target in the management of pancreatic malignancies. A similar association is seen in salivary carcinomas. Accentuated Skp2 expression is associated with decreased survival probability rates and a worse clinical outcome (Ben-Izhak et al., 2009).

Similarly, Skp2 has a role to play in the etio-pathogenesis of lung carcinomas. Non-small cell lung cancers typically demonstrated increased Skp2 expression. It promotes cellular invasion in pulmonary malignancies by up regulating matrix metalloproteinase-9 (MMP-9) as well as matrix metalloproteinase-2 (MMP-2; Hung et al., 2010). Not surprisingly, agents such as tubocapsanolide A attenuate the expression of Skp2 and thereby have a negative effect on cellular proliferation in pulmonary malignancies (Chang et al., 2007). Interestingly, WIF1 causes inhibition of proliferation in tumors such as bladder carcinomas by modulating Skp2 function besides regulating the expression of c-myc (Tang et al., 2009).

Clearly, Skp2 is involved in the pathogenesis of a number of systemic malignancies. It is a potential onco-target and further studies are needed to further identify compounds that can target Skp2 expression.
